# Unmasking a neurotoxic duo: a comprehensive overview of fentanyl–xylazine crosstalk in kinase pathways

**DOI:** 10.3389/fncel.2026.1851956

**Published:** 2026-08-12

**Authors:** Sally N. Pauss, Zachary A. Kipp, Cassandra D. Gipson, Terry D. Hinds Jr

**Affiliations:** 1 Department of Pharmacology and Nutritional Sciences, Drug and Disease Discovery D3 Research Center, University of Kentucky College of Medicine, Lexington, KY, United States; 2 Barnstable Brown Diabetes Center, University of Kentucky College of Medicine, Lexington, KY, United States; 3 Markey Cancer Center, University of Kentucky, Lexington, KY, United States

**Keywords:** addiction, PamGene, PamStation, serine threonine kinase, substance use disorder, tyrosine kinase

## Abstract

**Introduction:**

Fentanyl is the main cause of the US overdose crisis, responsible for over half of overdose deaths, totaling around 75,000 lives each year. The contamination of the illegal drug supply with xylazine has been linked to an increase in overdose fatalities. Adding xylazine to fentanyl causes additional health issues, including tissue necrosis, more severe withdrawal symptoms, and higher overdose lethality, while also potentially reducing the effectiveness of naloxone. Therefore, understanding the molecular signaling events when fentanyl is combined with xylazine is crucial.

**Methods:**

In these experiments, we treated the human neuronal cell line SH-SY5Y with fentanyl and xylazine and conducted detailed analyses using the PamGene PamStation to measure kinase activities across over 500 pathways, thereby stratifying and identifying affected signaling pathways.

**Results:**

Our results show that fentanyl and xylazine impact kinase activity differently when either is used alone, and often, xylazine diminishes the kinase activity changes caused by fentanyl alone. We also found that the combination of these drugs uniquely increases the phosphorylation of diverse substrates compared with each drug alone.

**Discussion:**

These are the first kinome analyses performed on human cells with fentanyl and xylazine, which may uncover new therapeutic targets to counteract their harmful combined effects.

## 1 Introduction

The misuse of fentanyl has become an extensive global concern. Although overdose rates have diminished since 2024, over 70,000 deaths annually in the United States still result from fentanyl-related overdoses (Garnett et al., 2026). Fentanyl is an opioid that binds to the mu-opioid receptor (MOR) to produce analgesic and euphoric effects. Initially developed for clinical use in sedation and pain relief, these properties also contribute to its potential for addiction and illicit consumption. The adulteration of the illegal drug market with xylazine has been linked to a rise in overdose-related fatalities (Edinoff et al., 2024). The addition of xylazine to fentanyl introduces numerous additional adverse effects, such as tissue necrosis (Ciccarone et al., 2025), precipitated withdrawal (Strickland et al., 2026; Sadek et al., 2024), and increased lethality in overdose cases, while potentially reducing the effectiveness of naloxone (Acosta-Mares et al., 2023). Therefore, it is crucial to understand the signaling differences that occur when fentanyl is combined with xylazine.

The MOR is a G protein-coupled receptor (GPCR) activated by ligands including endorphins and opioids. The MOR is coupled to inhibitory G proteins, Gα_*i*_ and Gα_*o*_, but can also induce dopaminergic reward signaling. Following activation of MOR, Gα_*i*_ inhibits adenylyl cyclase, causing a decrease in protein kinase A (PKA) activity and cAMP response element-binding protein (CREB) activation (Swingler et al., 2025). Another major effector of opioid and MOR signaling is the recruitment of β-arrestin to the GPCR. β-arrestin facilitates receptor desensitization and internalization (Swingler et al., 2025). Through less clearly defined mechanisms, kinases such as the mitogen-activated protein kinase (MAPK) family (ERK, JNK, p38) are activated downstream of G proteins and β-arrestin. Research has demonstrated that various MOR ligands can induce differential phosphorylation at specific sites on the MOR, suggesting activation of distinct kinase pathways (Doll et al., 2011). Fentanyl also induces hypoxic stress that may contribute to neurodegeneration, with kinases such as PDK1, p38, GSK-3β, and more being implicated (Al-Ameer et al., 2026).

Xylazine is a veterinary anesthetic that functions as an agonist at the α2-adrenergic receptor, inhibiting the release of epinephrine and norepinephrine, thereby inducing sedation and analgesia. Its use can result in decreased heart rate and respiration, thereby elevating associated risks. Limited information is available regarding the effects of xylazine in humans, as it has not received approval from the Food and Drug Administration (FDA) for human use. The first documented case of xylazine abuse occurred in Puerto Rico in 2012, and since then, illicit drug manufacturers have incorporated it (Reyes et al., 2012). Findings from 2023 indicate that xylazine is detected in nearly 100% of illicit fentanyl samples (The Center for Forensic Science Research & Education, 2023). This phenomenon has prompted increased research into xylazine and its combination with other substances. Studies indicate that the half-life of xylazine in humans exceeds previous animal-based estimates, reaching approximately 12 h (Lin et al., 2025). Recent research demonstrates that xylazine acts as a full agonist at the kappa opioid receptor and also binds to the serotonin 7 receptor (5-HT_7_R) (Bedard et al., 2024).

The combination of fentanyl and xylazine has been shown to increase the mortality associated with these substances (Acosta-Mares et al., 2023; Smith et al., 2023). A sublethal dose of fentanyl was observed to reduce the LD_50_ of xylazine by a factor of five, whereas xylazine decreased the LD_50_ of fentanyl by a factor of one hundred (Smith et al., 2023). Research indicates that the addition of xylazine diminishes self-administration of fentanyl (Khatri et al., 2024). Individuals who have used opioids in conjunction with xylazine have reported experiencing heightened sedative effects; however, this combination also results in notably more severe withdrawal symptoms (Reed et al., 2025). Many drug users have expressed a reluctance to use xylazine (Reed et al., 2022). Murine studies are presently confirming that xylazine is not reinforcing when administered independently and does not enhance fentanyl-induced conditioned place preference, implying that it does not augment its rewarding effects (Acosta-Mares et al., 2023). Recent studies have shown that fentanyl- and xylazine-treated cells exhibit increased JAK2 and PI3K expression, and molecular docking suggests that fentanyl and xylazine may bind to these kinases (Wang et al., 2026). Despite these scientific advances, the understanding of how the incorporation of xylazine influences fentanyl-induced signaling remains limited.

In these experiments, the human neuronal cell line SH-SY5Y was treated with fentanyl and xylazine, and kinase activity was comprehensively analyzed using the PamGene PamStation technology. The results indicated that fentanyl prompted phospho-tyrosine kinase (PTK) activity while concurrently suppressing serine/threonine kinase (STK) activity. Conversely, xylazine exhibited predominantly inhibitory effects on kinase activity. The combined application of fentanyl and xylazine demonstrated a general attenuation of fentanyl-induced alterations, notably exerting an inhibitory influence on STK kinases. These findings elucidate signaling pathways modified by fentanyl, xylazine, and their combination, thereby contributing to the identification of potential novel therapeutic targets aimed at mitigating the adverse effects linked to their concurrent usage.

## 2 Materials and methods

### 2.1 Cell culture and treatments

The neuroblastoma cell line SH-SY5Y, derived from a female patient, was utilized in these investigations owing to its catecholaminergic characteristics, including dopamine signaling (Xicoy et al., 2017). The cells were cultured in Dulbecco’s Modified Eagle Medium (DMEM) supplemented with 10% fetal bovine serum and 1% antibiotics-antimycotics and subjected to a 4-h treatment. The treatments included vehicle DMSO at 0.1% concentration for all treatments, RNase-free water, 10 nM fentanyl, 100 μM xylazine, and a combination of 10 nM fentanyl with 100 μM xylazine. These concentrations and time frame were chosen based on previous literature utilizing the same or similar cell lines with these treatments (Daijo et al., 2011; Park et al., 2013). Subsequently, the cells were harvested for subsequent analysis.

### 2.2 PamGene PamStation sample preparations

Protein was extracted using the Mammalian Protein Extraction Reagent (MPER) (Thermo Fisher Scientific, CAT #78503), Halt Phosphatase Inhibitor (Thermo Fisher Scientific, CAT #78503), and Protease Inhibitor Cocktail (Sigma, CAT #P2714). Protein concentration was quantified by measurement with the Pierce BCA Protein Assay Kit (Sigma, CAT #P2714) and measured in triplicate. Three biological replicates per treatment group were applied to the PamChips. 5 μg of protein from each sample was used for PTK analysis, and 1 μg was used for STK analysis. The PTK and STK PamChip assays were carried out on the PamStation12 (PamGene International, ‘s-Hertogenbosch, Netherlands), following our previously described procedures (Lee et al., 2025, 2026; Bates et al., 2023, 2026; Creeden et al., 2022). Phosphorylation of 196 PTK and 144 STK substrates was quantified using fluorescently labeled antibodies, as previously described (Kipp et al., 2026; Bates et al., 2024).

### 2.3 Kinome data bioinformatic analysis

Images captured by the PamStation, showing fluorescently tagged phosphorylation across the arrays, were exported and analyzed using Tercen BioNavigator software. Images of each PamChip taken at progressively longer exposure durations were used to fit linear models to the signal intensity. Spots demonstrating non-linear increases in signal intensity were excluded. The fold change in signal intensity was determined for each phospho-peptide, and these values were averaged across three biological replicates. Differential phosphorylation was identified (Creeden et al., 2022). Upstream kinase activities responsible for phosphorylation variations were identified using the BioNavigator Upstream Kinase Analysis (UKA) software from PamGene and the Kinome Random Sampling Analyzer (KRSA) package (DePasquale et al., 2021). MEOW plots were generated employing KRSA’s Log2FC of kinase substrates mapped to the respective kinase multiplied by the delta confidence. Delta confidence was ascertained through KRSA random sampling analysis peacock plots, by dividing the number of substrates mapped to the specified kinase identified in the experimental group by the average number of substrates expected through random sampling iterations, as previously described {formula: [Log2 fold change (FC) of kinase substrates * (multiplied by) Δ confidence (experimental hits/mean hits of 2,000 random sampling iterations)]} (Kipp et al., 2026). PANDI plots were produced utilizing the BioNavigator median kinase statistic (MdKS) to illustrate changes in kinase activity. MdKS represents the direction and magnitude of the change in kinase activity between the treatment and control. The z scores are computed in R using KRSA. Using the observed substrates (number of hit peptides mapped to the kinase), sampling average (average number of peptides mapped to that kinase across 2,000 random sampling iterations), and standard deviation, the z score is calculated [Z = (Observed–SamplingAvg)/SD]. This is calculated over the three chips and using three LFC significance thresholds, 0.2, 0.3, and 0.4. This data is then used to generate the plots, with the large dot indicating the average.

### 2.4 PamGene BioNavigator statistical analysis

Data analysis was conducted utilizing Tercen BioNavigator, incorporating both phosphosite and upstream kinase analyses. PamGene’s BioNavigator software automatically implements multiple testing correction (MTC) when calculating differential substrate phosphorylation between treated groups and controls, and applies an analysis of variance (ANOVA), followed by Dunnett’s *post-hoc* test for multiple comparisons to identify significant differences among each test condition. The upstream kinase identification was executed using BioNavigator UKA (PamGene). The UKA analysis identifies the kinases most likely responsible for phosphorylation events on the PamChip over time, using substrates that have passed quality control measures. BioNavigator employs a mapping file that contains both experimentally confirmed and predicted substrates for each kinase, ranked by confidence from 0 (high confidence based on *in vitro* or *in vivo* experiments) to 12 (low confidence based on predictive models). The UKA algorithm generates scores used to rank the responsible kinases, including median kinase statistic (represents the log fold change showing directionality and magnitude of change), significance score (uses permutation tests where the samples are permuted to represent the difference between the kinase statistic of the actual sample versus that of the permuted samples and denotes differences between the control and treatment), specificity score (uses permutation tests where the peptides are permuted to represent the difference between the kinase statistic of the actual sample versus that of the permuted samples and denotes differences observed are not random), and the median final score (the sum of the significance and specificity score and utilizes a cut-off of 1.3 to denote significance and is shown in the volcano plots).

### 2.5 CORAL phylogenetic trees

The phylogenetic trees were created to show the activity of the entire kinome for a given comparison, using the median kinase statistic (kinase activity) and the median final score (significance) from BioNavigator. The final score from BioNavigator is determined by the sum of the significance score, which indicates the probability that a kinase is differentially active between conditions, and the specificity score, which indicates the probability that the effect could not have occurred randomly. Data was compiled for both PTK and STK for each comparison. The CORAL R package was utilized to visualize the trees, as previously described (Creeden et al., 2022). The node color reflects kinase activity, whereas the size indicates confidence.

### 2.6 Cytoscape human brain kinome network analysis

To generate networks, the top and bottom 25 kinases were used to capture hypoactive and hyperactive kinases, sorted by median final score, and compiled into PTK and STK to create the network nodes and edges. This software was built from gene-matrix transposed files downloaded from KEA3 and filtered to include genes highly expressed in the human brain, as identified by mRNA expression consensus data from the Human Protein Atlas. The network edges were determined by compiling kinase interactions supported by multiple datasets and weighting them by the number of datasets supporting each interaction. The network was visualized using Cytoscape.

## 3 Results

### 3.1 Phospho-tyrosine kinase (PTK) analysis

**FIGURE 1:**
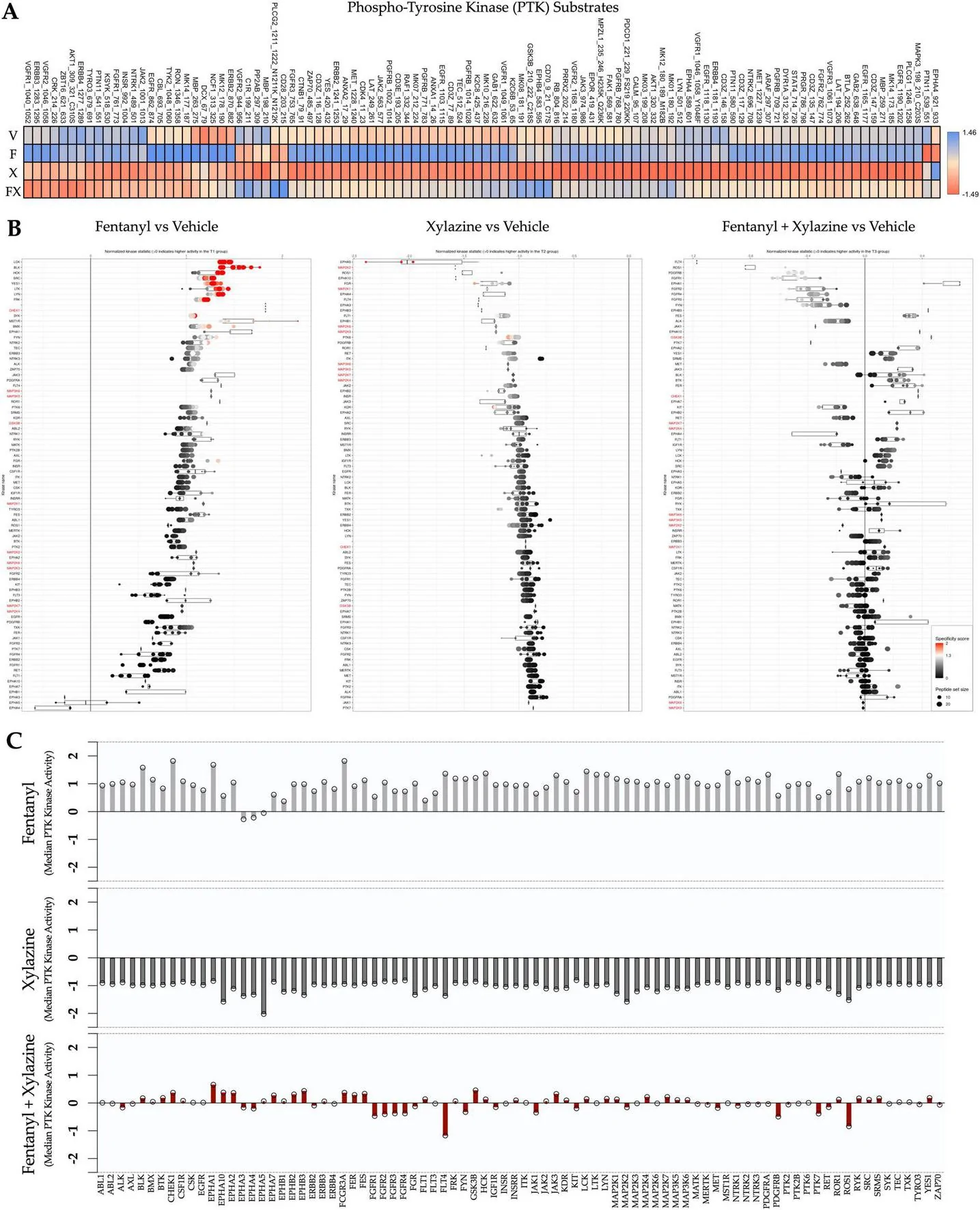
Assessment of phospho-tyrosine kinase (PTK) families. **(A)** The heatmap depicts substrate phosphorylation on the PTK PamChip across all treatments. **(B)** The waterfall plots, generated by BioNavigator, illustrate PTK kinase activity by displaying the median kinase statistic for each kinase relative to the vehicle. They are ranked according to the final score, which serves as a measure of confidence. The red dots indicate higher specificity, and the red text on the kinase names signifies that the kinase can be classified as either a PTK or STK. **(C)** The PANDI plots demonstrate kinase activity by displaying the median kinase statistic for each kinase across all treatments compared to the vehicle, with kinases sorted alphabetically to facilitate alignment across treatments. V, vehicle; F, fentanyl; X, xylazine; FX, fentanyl and xylazine.

To identify the kinase pathways influenced by fentanyl and xylazine, SH-SY5Y cells were subjected to treatments with fentanyl and xylazine individually, as well as in combination. The activities of phospho-tyrosine (PTK) and serine/threonine kinases (STK) were assessed utilizing PamGene PamStation kinome technology. Figure 1A shows the substrate phosphorylation of PTK substrates with each treatment. Based on the heatmap, fentanyl increased phosphorylation overall, whereas xylazine decreased it compared to the vehicle. The combination treatment had dichotomous effects on PTK substrate phosphorylation compared with either treatment alone. Interestingly, a group of 6 substrates was phosphorylated more by the combination treatment than by either treatment alone (CD28, PLCG2, MBP, PP2AB, C1R, and VGFR2). CD28 and PLCG2 were also more phosphorylated with the combination of fentanyl and xylazine compared to the vehicle, and both have been implicated in immune and calcium signaling (Yang et al., 2017; Jackson et al., 2021). Multiple bioinformatics tools were employed to trace modifications in substrate phosphorylation back to their corresponding kinases, thereby assessing kinase activity. Figures 1B,C depict PTK kinase activity, which was determined from the phosphorylation data presented in Figure 1A. Specifically, Figure 1B displays waterfall plots generated by BioNavigator, organized by confidence level, whereas Figure 1C illustrates PANDI plots that classify kinase activity alphabetically. Fentanyl caused an overall increase in PTK kinase activity, while xylazine caused a suppression in kinase activity compared to the vehicle. The combination of fentanyl and xylazine resulted in overall smaller magnitude changes with a dichotomy, in which some kinases were more active, and some were less active, but to a lower magnitude than the changes seen with either individual treatment. According to the waterfall and PANDI plots, the top fentanyl-induced kinases are LCK, BLK, and HCK (Figure 1C). The top kinases suppressed by xylazine are EphA5, MAP2K2, and ROS1. The top changed kinases for the combination treatment are suppression of FLT4, ROS1, and PDGFRβ, while EPHA1 was the top increased kinase. Figure 1C facilitates easy identification of differential effects between treatments and highlights the blunted treatment-induced changes in kinase activity observed in the combination treatment.

**FIGURE 2:**
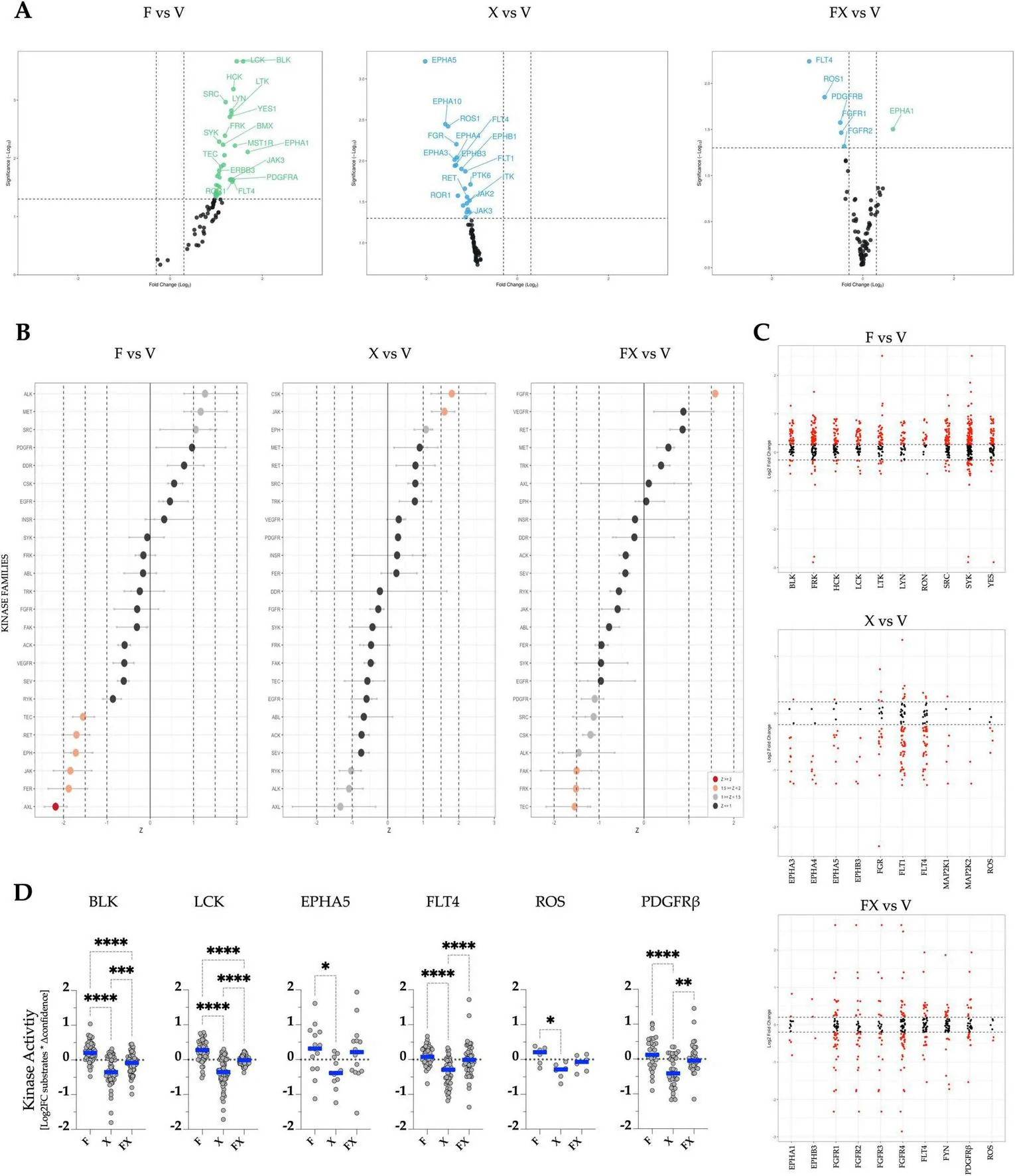
Evaluation of the phospho-tyrosine kinase (PTK) individual pathways. **(A)** Volcano plots generated by BioNavigator illustrate the Log2 fold change for each kinase relative to the vehicle, highlighting those surpassing the significance threshold indicated by the dotted line. Kinases exhibiting significantly increased activity are denoted in green, whereas those with significantly decreased activity are shown in blue. **(B)** Z-score plots produced by KRSA demonstrate the overall activity of kinase families within the treatment comparison. Red dots indicate families that have exceeded the significance threshold. **(C)** Reverse KRSA plots present the most altered kinases in each comparison, with each column showing dots displaying the log2 fold changes in kinase substrate phosphorylation relative to the vehicle for all of the kinases’ substrates. **(D)** MEOW plots depict kinase activity and confidence, illustrating each substrate’s fold change multiplied by the change in confidence of that kinase within the comparison, with the blue line representing the mean. Significance levels are indicated as follows: *p < 0.05, **p < 0.01, ***p < 0.001, ****p < 0.0001. V, vehicle; F, fentanyl; X, xylazine; FX, fentanyl and xylazine.

Figure 2A presents volcano plots showing kinase activity changes relative to the vehicle for each treatment, highlighting kinases with significant changes. It is evident once again that fentanyl and xylazine induce contrasting effects on overall kinase activity, and these effects are diminished in the combined treatment, with only a limited number of kinases surpassing the threshold for significant change compared to the vehicle. Figure 2B displays Z-score plots ranking kinase families by their activity change relative to the vehicle. Kinase families such as AXL, FER, JAK, EPH, RET, and TEC were all significantly decreased following fentanyl administration. Conversely, the CSK and JAK kinase families exhibited increased activity in response to xylazine. In the combined treatment, the FGFR family showed increased activity, whereas TEC, FRK, and FAK demonstrated decreased activity. Figure 2C depicts the most significantly altered individual kinases as identified by KRSA for each comparison. Notably, many of these kinases align with those identified by BioNavigator’s waterfall plots, thereby reinforcing the validity of the kinase activity assessments. Figure 2D presents MEOW plots for six of the most prominently affected kinases across the three treatment conditions, as seen by their appearance in Figure 2C. The activities of BLK and LCK differed significantly among these treatments. Additionally, the activities of FLT4 and PDGFR were reduced by xylazine but subsequently increased to approximately the same extent by either fentanyl alone or the combination of fentanyl and xylazine.

### 3.2 Serine-threonine kinase (STK) analysis

**FIGURE 3:**
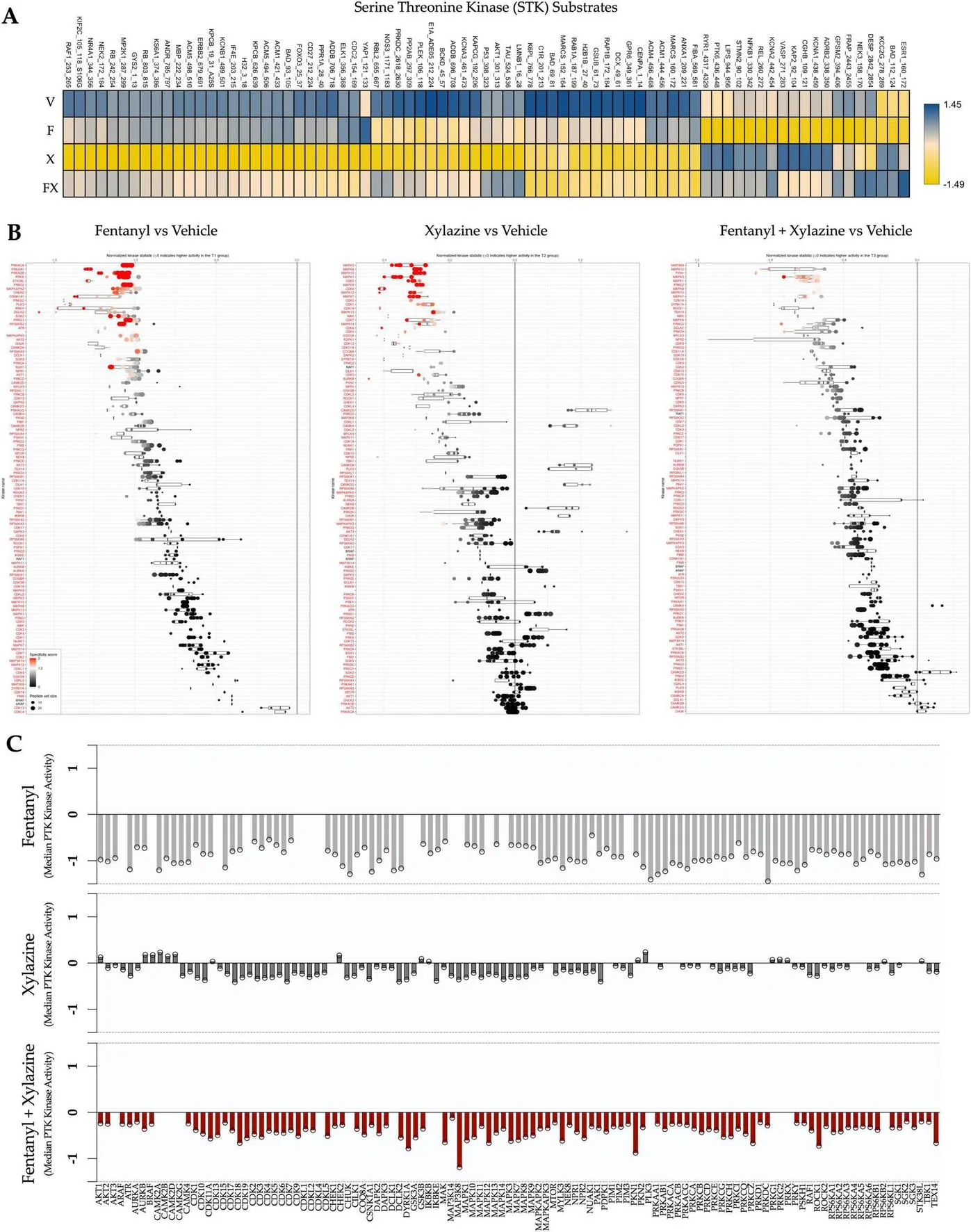
Evaluation of serine-threonine kinase (STK) families. **(A)** This heatmap illustrates the phosphorylation of each substrate on the STK PamChip across each treatment. **(B)** The waterfall plots were generated by BioNavigator and demonstrate STK kinase activity by displaying the median kinase statistic for each kinase relative to the vehicle. They are ranked by the final score, which is a measure of confidence. The red color dots indicate higher specificity, and the red text on the kinase names indicates that the kinase can be classified as either a PTK or STK. **(C)** The PANDI plots show kinase activity by displaying the median kinase statistic for each kinase across all treatments compared to the vehicle, and are sorted alphabetically to align kinases across treatments. V, vehicle; F, fentanyl; X, xylazine; FX, fentanyl and xylazine.

The STK assay was analyzed similarly to the PTK assay. Figure 3A shows the phosphorylation of the STK substrates in response to vehicle, fentanyl, xylazine, or the combination. Fentanyl and xylazine individual treatments had less consistent phosphorylation changes on STK substrates than on PTK substrates. Some substrates were hyperphosphorylated by the combination treatment, but hypo-phosphorylated by either treatment alone compared to the vehicle. These proteins are DESP, NEK3, LMNB1, TAU, PLEK, PP2AB, NOS3, and RBL2. Many of these proteins are involved in cytoskeletal remodeling and cell cycle regulation.

**FIGURE 4:**
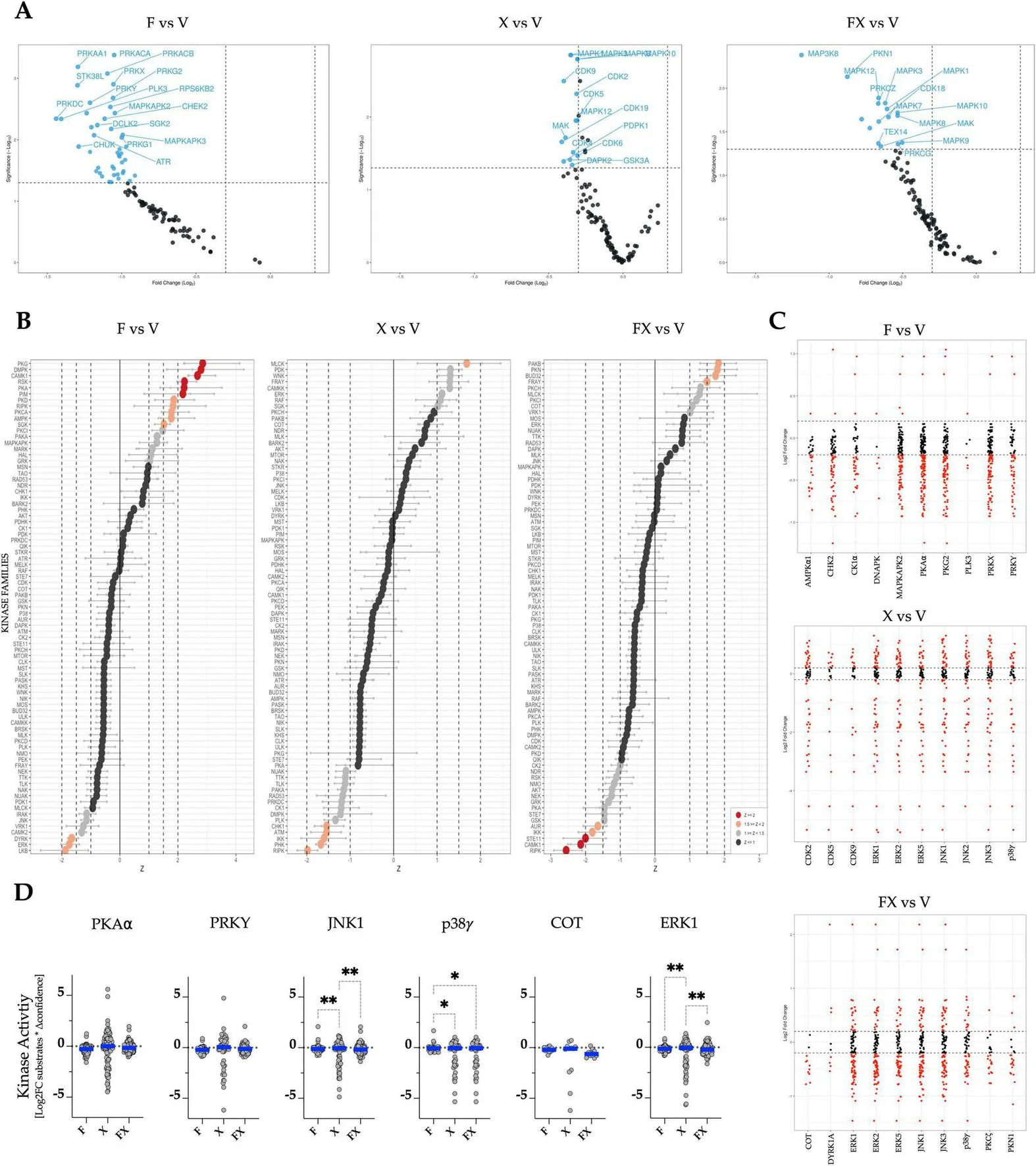
Serine-threonine kinase (STK) individual pathway assessment. **(A)** Volcano plots generated by BioNavigator depict the Log2 fold change for each kinase relative to the vehicle, with those surpassing the significance threshold indicated by the dotted line. Kinases exhibiting significantly increased activity are shown in green, whereas those with significantly decreased activity are shown in blue. **(B)** Z-score plots produced by KRSA illustrate the overall activity of kinase families within the treatment comparison. Red dots signify that the respective family has exceeded the significance threshold. **(C)** Reverse KRSA plots present the most significantly changed kinases in each comparison. Each column displays the substrate phosphorylation Log2 fold changes for all kinases. **(D)** MEOW plots visualize kinase activity and confidence levels, illustrating each substrate’s fold change multiplied by the corresponding change in confidence for that kinase within the comparison. The blue line denotes the mean, with asterisks indicating significance levels: *p < 0.05, **p < 0.01. V, vehicle; F, fentanyl; X, xylazine; FX, fentanyl and xylazine.

Upstream kinase activity was assessed by alterations in substrate phosphorylation, and the STK kinase activity waterfall and PANDI plots are shown in Figures 3B,C. Contrary to the trend observed in the PTK analysis, fentanyl reduced the overall activity of STK kinases, while xylazine had mixed effects. The combination treatment also generally lowered kinase activity, suggesting that adding xylazine to fentanyl lessened the kinase activity suppression caused by fentanyl. Top changed kinases were as follows: fentanyl decreased activity of PRKACA, PRKAA1, and PRKACB; xylazine decreased activity of MAPK3, MAPK8, and MAPK10; and the combination decreased activity of MAP3K8, MAPK12, and PKN1. The data are also shown in volcano plots in Figure 4A, indicating significant changes in kinase activity for each comparison and highlighting the overall suppression of STK kinase activity by these three treatments. Figure 4B illustrates the activity levels of STK kinase families ranked by z-score. Although overall activity remained reduced compared to the control, kinase families such as PKG and DMPK were the most active in fentanyl-treated cells. The MLCK kinase family exhibited the highest activity in the xylazine treatment. Furthermore, the PAKB family demonstrated the greatest activity in the combined fentanyl and xylazine treatment. Figure 4C delineates the most significantly altered kinases in each comparison as determined by KRSA analysis. Notably, both KRSA and BioNavigator analyses identified several of the same kinases as exhibiting substantial changes following treatments. The kinase most suppressed by the combination therapy, MAP3K8/COT, was ranked highly in both analyses. Using six of the most significantly altered kinases identified via BioNavigator and KRSA, MEOW plots were produced to depict kinase activity levels, considering the confidence associated with each kinase’s change. The kinase activities of JNK1, p38, and ERK1 were significantly different across treatments, as evidenced by the MEOW plots.

### 3.3 Additional comparisons

**FIGURE 5:**
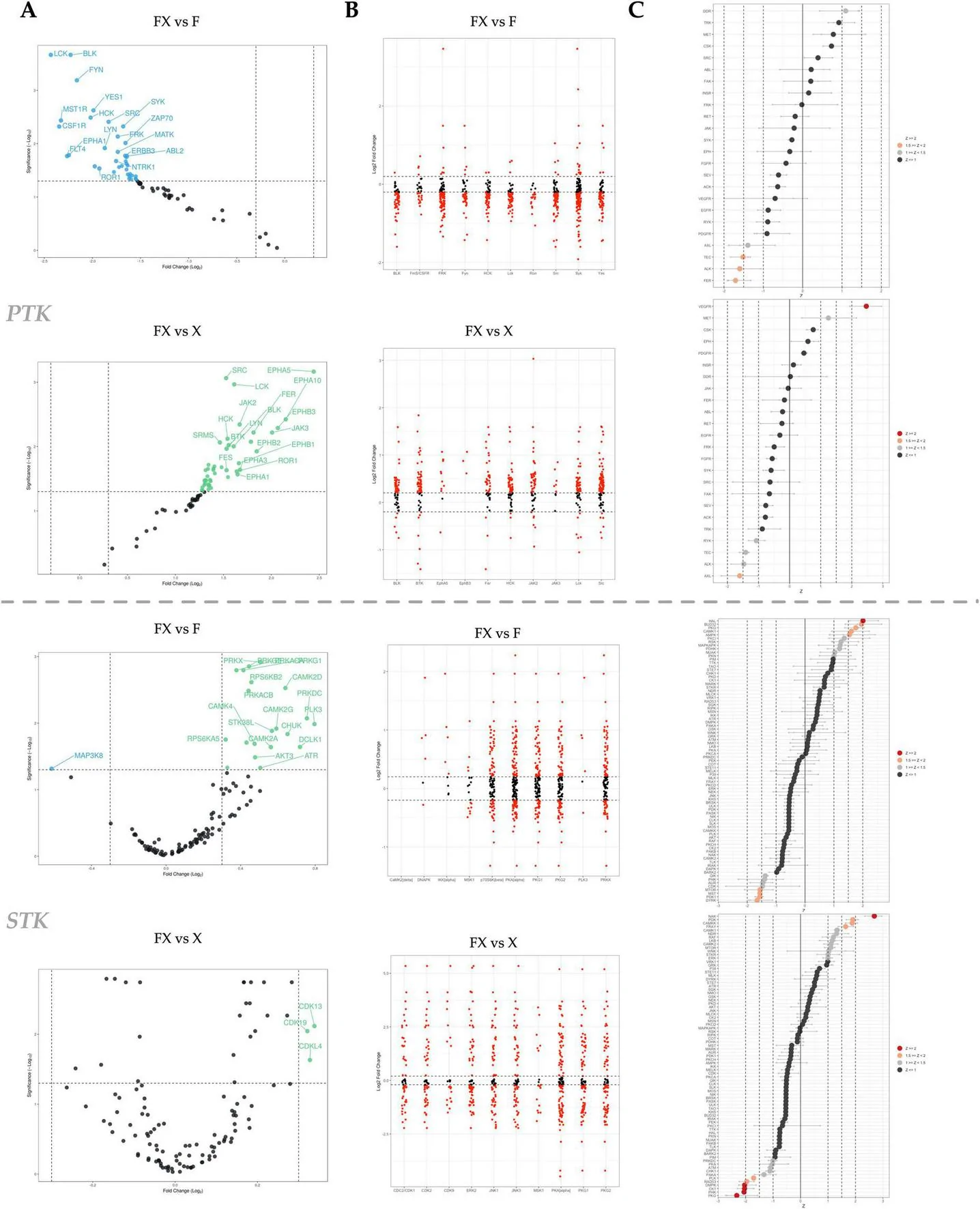
Comparative analysis of PTK and STK activity. The data were analyzed to evaluate the effects of fentanyl and xylazine relative to each agent used independently. **(A)** Volcano plots, generated via BioNavigator, illustrate the Log2 fold change for each kinase in relation to the vehicle, with those surpassing the significance threshold indicated by the dotted line. Kinases exhibiting significantly increased activity are marked in green, whereas those with significantly decreased activity are marked in blue. **(B)** Reverse KRSA plots depict the top kinases with altered activity in each comparison. Each column represents the log2 fold changes in substrate phosphorylation across all kinases. **(C)** Z-score plots, produced through KRSA, display the overall activity levels of kinase families within each treatment comparison. Red dots denote families that exceed the significance threshold.

To directly compare alterations in kinase activity in the combination, we analyzed PTK and STK activities of fentanyl and xylazine relative to their respective activities alone. The volcano plots in Figure 5A demonstrate that the PTK kinase activity experienced a more pronounced change compared to the STK kinase activity across these comparisons. The combination of fentanyl and xylazine resulted in a decrease in PTK kinase activity relative to fentanyl alone and an increase relative to xylazine alone. Conversely, STK activity was predominantly elevated with the combination treatment compared to either individual treatment. Figure 5B presents the reverse KRSA plots for each comparison, highlighting the kinases with the most significant alterations. Figure 5C displays the z-scores of kinase activity for each treatment, highlighting the kinases with the highest and lowest activity.

### 3.4 Phylogenetic trees and network analysis

**FIGURE 6:**
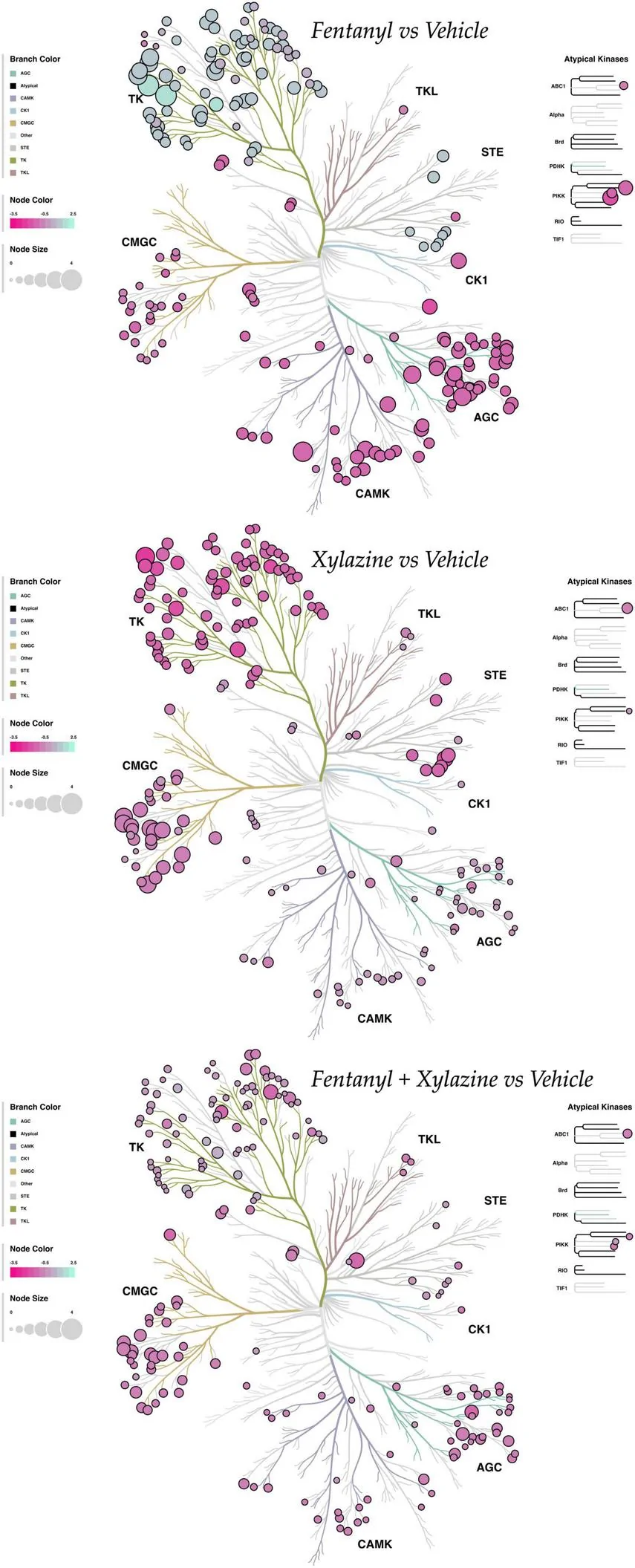
Kinome phyla comparisons. Phylogenetic trees illustrate variations in kinase activity throughout the entire measured kinome. The color of each node signifies the kinase activity derived from the median kinase statistic. The size of each node reflects the confidence level, as indicated by the final score.

Figure 6 presents phylogenetic trees illustrating kinase activity across the entire measured kinome for each treatment comparison. The trees indicate that fentanyl induces greater PTK activity than the other treatments. These substantial increases in kinase activity are abolished with the addition of xylazine, as evidenced by the disappearance of the prominent blue-green circles from fentanyl to fentanyl plus xylazine. Overall, STKs were similarly regulated across treatments, with many kinases in the class showing decreased activity.

**FIGURE 7:**
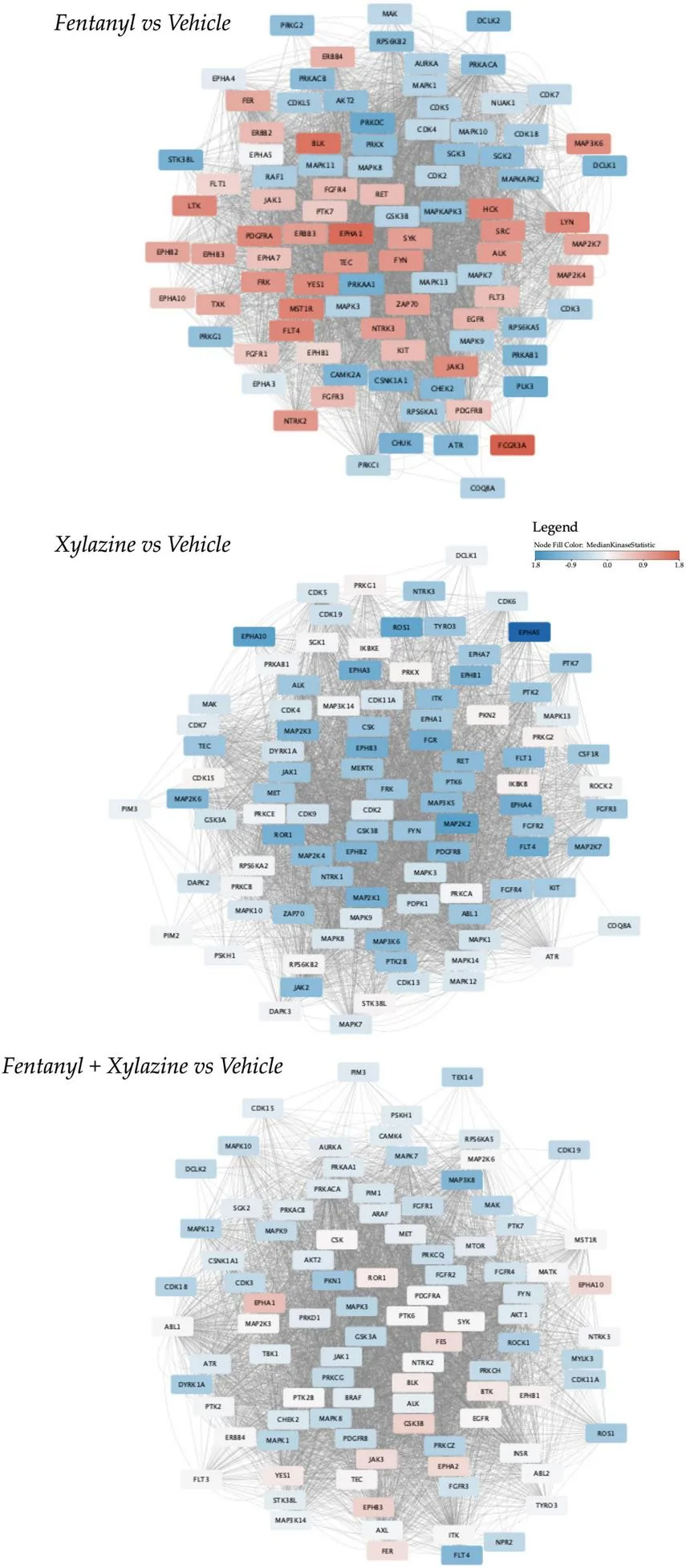
Cytoscape human brain kinome network analysis. Networks depict the top and bottom 25 PTK and STK kinases identified by BioNavigator UKA, filtered by genes expressed in the brain. The color of the nodes signifies the kinase activity relative to the control.

Figure 7 shows the top and bottom 25 kinases for each comparison, filtered to only include genes expressed in the human brain. The kinases are connected by lines indicating interactions confirmed by multiple databases. As the node color indicates kinase activity, the fentanyl treatment caused the greatest increase in kinase activity. The xylazine treatment caused an overall decrease in kinase activity, as indicated by the blue color. The combination treatment shows some increases and some decreases in activity. This network also confirms that many of the main targets identified within the treatments are expressed in the human brain.

## 4 Discussion

This study demonstrates that the combination of fentanyl and xylazine results in distinct alterations in protein phosphorylation and kinase activities compared to either treatment administered individually. Prior research using the same cell line has indicated that fentanyl elevates angiogenic markers, including hypoxia-inducible factor 1-alpha (HIF-1α) and vascular endothelial growth factor (VEGF), effects that are reversible with naloxone (Daijo et al., 2011). As opioid signaling is believed to involve downstream kinase pathways, inhibitors targeting MAPK, PI3K, and PKC were employed, all of which impeded fentanyl-induced HIF-1α expression, implying that these kinases operate upstream of HIF-1α in fentanyl signaling (Daijo et al., 2011). Additionally, it was determined that this signaling cascade is associated with opioid-induced reactive oxygen species production (Daijo et al., 2011). Our investigation, employing a comparable dosage and a shorter time course, revealed that fentanyl generally suppressed PKC and MAPK activity while augmenting MAP2K activity, suggesting the presence of a negative feedback mechanism. Conversely, proteins implicated in endothelial cell migration and angiogenesis, such as VEGF and NOS3, exhibited increased phosphorylation in response to the combined fentanyl and xylazine treatment. Other research has demonstrated that fentanyl suppresses the activity of Akt1 and CAMK2A, findings that our study corroborated (Zheng et al., 2010; Li et al., 2020). The kinase families discussed—including PKC, PKA, ERK, and MAPK—have also been implicated in dopaminergic signaling, which is critical for the rewarding effects of drugs (Gurevich et al., 2016). Both CDK5 and PKC are reported to attenuate dopaminergic signaling (Chergui et al., 2004; Zhang et al., 2007). It was observed that both fentanyl alone and its combination with xylazine inhibited these kinases’ activity, potentially amplifying dopaminergic signaling. Nonetheless, these effects should be interpreted in conjunction with the activity of other kinases and regulatory elements involved in dopaminergic signaling.

Our research has identified multiple substrates hyperphosphorylated by fentanyl and xylazine involved in cytoskeletal remodeling. Upstream analysis highlighted several altered kinases, visualized as MEOW plots. Among these, MAP3K8 (TPL2/COT) stands out as an interesting potential mediator for opioid and fentanyl signaling, as it is part of innate immune and inflammatory signaling pathways (Hedl and Abraham, 2016). These pathways are increasingly linked to opioid dependence, withdrawal, hyperalgesia, and relapse. Addiction is now understood as a complex disease involving multiple systems including glial activation, cytokine signaling, and neuroimmune remodeling, which can reinforce compulsive behavior and worsen withdrawal symptoms (Uhl et al., 2019; Kosten and George, 2002; Hedl and Abraham, 2016). EPHA5 is another key candidate due to its roles in axon guidance, synapse formation, and circuit plasticity, critical processes in rewiring reward and stress circuits implicated in addiction; however, direct opioid-specific evidence remains limited (Uhl et al., 2019; Levis et al., 2021). PDGFRβ is also significant as an indirect candidate because it plays a role in pericyte function, vascular health, and maintaining the blood-brain barrier, which may be relevant in cases of severe fentanyl toxicity or repeated exposure (Gamble et al., 2022; Volkow and Blanco, 2021).

In contrast, LCK and BLK are primarily recognized as immune-cell kinases rather than addiction-related kinases, so their presence in an opioid dataset likely indicates immune activity or effects related to cell-type composition, rather than a direct role in reward behavior. EPHA1 and FLT4 (VEGFR3) are only broadly plausible in mechanisms involving cell-cell signaling, inflammation, or neurovascular remodeling. ROS1 has minimal established connections to addiction biology. Overall, this list does not resemble a typical mu-opioid receptor signaling panel; it seems enriched for pathways related to neuroimmune responses, structural plasticity, and vascular responses, aligning with current views of opioid and fentanyl addiction as disorders involving both reward pathways and inflammatory adaptation (Uhl et al., 2019; Gamble et al., 2022). It may also indicate pathological neuronal remodeling, leading to decreased synaptic plasticity and neurodegeneration. In neurons, this could relate to neuroplasticity and learning (Priel et al., 2010). Further research is needed to understand how the observed kinase activity changes influence neuronal remodeling and pathology. In other tissues, the hyperphosphorylated proteins in our study are linked to wound healing (Ahangar et al., 2022). Given the necrotic wounds associated with xylazine use in humans, these findings could prompt additional investigation into these treatments in non-neuronal cells, such as epidermal cells.

The addition of xylazine to illicit drug supplies has heightened concerns for public health, as rates of overdose and hospitalization related to substance use have increased. More recently, a more potent α2-adrenergic agonist, medetomidine, has been introduced into illicit drug supplies. Reportedly, medetomidine is 100–300 times more potent than xylazine and has begun replacing xylazine in drug adulteration practices (Philadelphia Department of Public Health, 2025). Similar to xylazine, it is reported to cause significantly more severe withdrawal symptoms. The Philadelphia Department of Public Health reports a 307% increase in substance use-related skin and soft tissue injuries, likely attributable to the introduction of xylazine into drug supplies (Philadelphia Department of Public Health, 2025). Interestingly, these rates have since decreased following the introduction of medetomidine, although visits for withdrawal have continued to rise (Philadelphia Department of Public Health, 2025). While our studies primarily focus on xylazine, they may offer insights into drugs with similar mechanisms of action, such as medetomidine.

One limitation of our study is the use of the SH-SY5Y cell line. This cell line is derived from a 4-year-old female patient and has some properties of catecholaminergic neurons (Kovalevich and Langford, 2013). These cells have also been shown to express the mu-opioid receptor, and its expression level is responsive to agonists such as methadone (Saify and Saadat, 2016; Daijo et al., 2011). However, their translatability to the human brain may be limited. While various other studies have utilized this cell line to study the effects of fentanyl, like any model, it has its limitations (Daijo et al., 2011; Wang et al., 2026). The findings of this study could be built upon by using an animal model of drug intake, which would also allow evaluation of the effects of drug metabolites. Another limitation is that the present studies rely solely on the PamStation, which measures kinase activity by changes in phosphorylation. Future studies may explore functional validation of kinases with kinase inhibitors.

Overall, the understanding of fentanyl signaling in addiction remains incomplete. Even more elusive is the manner in which xylazine modifies these pathways. To our knowledge, this is the first study to utilize the PamStation kinase technology to examine the activities of hundreds of kinases in response to fentanyl and xylazine treatments. In this context, we have presented a comprehensive analysis of alterations in kinase activity induced by fentanyl and xylazine in human cells (Wang et al., 2026). These findings begin to contribute to addressing the ongoing public health crisis related to the adulterated illicit drug supply.

## Data Availability

The datasets presented in this study can be found in online repositories. The names of the repository/repositories and accession number(s) can be found below: https://doi.org/10.6084/m9.figshare.32782569.
